# Integrative multi-omic profiling in blood reveals distinct immune and metabolic signatures between ACPA-negative and ACPA-positive rheumatoid arthritis

**DOI:** 10.3389/fimmu.2025.1667662

**Published:** 2025-10-29

**Authors:** Benjamin Hur, Vinod K. Gupta, Minsik Oh, Hu Zeng, Cynthia S. Crowson, Kenneth J. Warrington, Elena Myasoedova, Vanessa L. Kronzer, John M. Davis, Jaeyun Sung

**Affiliations:** ^1^ Division of Computational Biology, Department of Quantitative Health Sciences, Mayo Clinic, Rochester, MN, United States; ^2^ Microbiomics Program, Center for Individualized Medicine, Mayo Clinic, Rochester, MN, United States; ^3^ School of Software Convergence, Myongji University, Seoul, Republic of Korea; ^4^ Division of Rheumatology, Department of Internal Medicine, Mayo Clinic, Rochester, MN, United States; ^5^ Department of Immunology, Mayo Clinic, Rochester, MN, United States; ^6^ Division of Clinical Trials and Biostatistics, Department of Quantitative Health Sciences, Mayo Clinic, Rochester, MN, United States; ^7^ Division of Epidemiology, Department of Quantitative Health Sciences, Mayo Clinic, Rochester, MN, United States

**Keywords:** biomarker discovery, multi-omic profiling, ACPA-negative and ACPA-positive rheumatoid arthritis, proteomics, metabolomics, plasma, machine learning

## Abstract

**Objective:**

To investigate whether patients with ACPA-negative (ACPA–) and ACPA-positive (ACPA+) rheumatoid arthritis (RA) exhibit distinct immune and metabolic profiles in blood, using integrative proteomic and metabolomic analyses. By uncovering subgroup-specific molecular signatures, we aim to improve the biological understanding of RA heterogeneity and support the development of more precise diagnostic and stratification strategies.

**Methods:**

We performed high-throughput proteomic and metabolomic profiling on plasma from a well-characterized cohort comprising 40 patients with ACPA– RA, 40 patients with ACPA+ RA, and 40 healthy controls. To identify key immune and metabolic differences, we applied statistical comparisons, pathway enrichment analyses, and network inference methods. Additionally, an integrative network-based machine learning framework was used to distinguish RA subgroups from controls based on plasma molecular profiles.

**Results:**

ACPA– and ACPA+ RA exhibited distinct plasma proteomic and metabolomic biomolecular signatures. Complement proteins (CFB, CFHR5, and F9) and the anti-inflammatory cytokine IL1RN were exclusively elevated in ACPA– RA and remained distinct in a treatment-naïve sub-cohort. Metabolomic analysis revealed subgroup-specific differences in lipid and pyrimidine metabolism, including contrasting patterns in bilirubin-derived metabolites. Correlation analyses identified differential associations between molecular features and clinical inflammatory markers across RA subgroups. An integrative machine learning framework incorporating multi-omic features achieved high classification performance in cross-validation (AUC ≥ 0.90), outperforming models based on single-omic data.

**Conclusion:**

This study suggests that ACPA status may not fully capture the biological heterogeneity between ACPA– and ACPA+ RA subgroups, indicating additional immune and metabolic distinctions that warrant further investigation. Our findings highlight the potential of multi-omic profiling to enhance RA diagnostics, refine disease stratification, and inform subgroup-specific disease management strategies.

## Introduction

Rheumatoid arthritis (RA) is a chronic autoimmune inflammatory disease that is diagnosed in nearly 5 per 1,000 adults worldwide (1–3). RA results in joint swelling, pain, deformities, bone erosion, and cartilage destruction (3–5). A key diagnostic marker for RA is the presence of anti-citrullinated protein autoantibodies (ACPA) in blood (6), with high specificity that exceeds 90% (4, 7, 8). However, the diagnostic sensitivity of ACPA-based tests for classifying RA cases is modest, ranging between 30–60% (8, 9). Often, RA can be clinically diagnosed even in the absence of circulating ACPA, a condition referred to as ACPA-negative RA (or ACPA– RA) (6). Importantly, as the absence of ACPA poses challenges in the diagnosis of early-stage RA, a delayed diagnosis can hinder the timely initiation of therapeutic interventions and increase the risk of joint damage (10, 11).

Traditionally, the primary distinction between ACPA– and ACPA+ RA has been considered to be this serological difference, with little attention given to other biological disparities. However, recent evidence suggests that these two RA subgroups may be fundamentally different in ways that extend beyond ACPA status alone. Recent studies suggest that ACPA– and ACPA-positive RA (or ACPA+ RA) represent distinct disease subgroups that differ in disease progression and treatment response (12, 13). These clinical disparities have prompted further investigations into the biomolecular differences between these subgroups utilizing high-throughput profiling techniques. For example, a genome-wide association study revealed significant differences in risk allele frequencies, mainly in the human leukocyte antigen (HLA) region, between ACPA– and ACPA+ RA (14). Research into the gut microbiome identified intestinal butyrate-metabolizing bacterial species associated with the presence of circulating ACPA (15). A serum autoantigen analysis, performed using liquid chromatography-tandem mass spectrometry (LC-MS/MS), uncovered subgroup-specific autoantigens and facilitated the development of classification panels for distinguishing ACPA– and ACPA+ RA (16). Furthermore, single-cell RNA sequencing of peripheral blood mononuclear cells (PBMCs) and synovial tissue revealed immune cell abnormalities unique to each RA subgroup, suggesting ACPA– RA may rely on different immune mechanisms and pathways (17). In our previous study, through multiplex autoantibody profiling of serum from patients with ACPA– and ACPA+ RA, we identified distinct IgG autoantibody repertoires for each subgroup (18).

Despite these landmark findings, no study has yet comprehensively and simultaneously examined the blood proteomic and metabolomic landscapes in ACPA– and ACPA+ RA. Proteomics provides a detailed map of the proteins driving cellular signaling pathways and systemic events in the circulatory system (19, 20), enabling us to observe immune responses in RA. Meanwhile, metabolomics explores the biochemical pathways that sustain cellular function, uncovering metabolic signatures shaped by intrinsic physiology, dietary factors, lifestyle, and external stimuli (21). By integrating blood proteomics and metabolomics, we can gain novel insights into the immune and metabolic processes specific to ACPA– and ACPA+ RA, as well as the coordinated mechanisms (e.g., enzymes and their substrates or products) through which proteins and metabolites influence disease onset and progression (22).

To address this critical knowledge gap, we performed global (untargeted) proteomic and metabolomic profiling on 120 individuals, comprising 40 patients with ACPA– RA, 40 patients with ACPA+ RA, and 40 healthy controls. Using controls as a reference point, we identified subgroup-specific differences in circulating immune and metabolic features, including complement proteins and cytokines. We also observed distinct correlation patterns between molecular features and clinical inflammation measures, suggesting potential differences in inflammatory regulation across RA subgroups. This high-resolution, multi-omic profiling study of circulating biomolecules not only deepens our understanding of the biological differences between these RA subgroups, but also introduces a novel strategy—incorporating machine learning—to inform the development of next-generation digital blood tests for RA.

## Materials and methods

### Study population, subject enrollment, and plasma sample collection

The study population consisted of patients with RA attending the outpatient practice of the Division of Rheumatology at Mayo Clinic in Rochester, MN, USA. Eligibility required patients to be adults 18 years of age or older with a clinical diagnosis of RA by a rheumatologist, fulfilling the American College of Rheumatology/European League Against Rheumatism 2010 revised classification criteria for RA (4). Patients were excluded if they did not comprehend English, were unable to provide written informed consent, or were members of a vulnerable population (e.g., incarcerated subjects).

RA was categorized into either ACPA– or ACPA+ RA subgroups based on the titer of anti-CCP antibodies detected by the Quanta Lite CCP3 IgG enzyme-linked immunosorbent assay (INOVA Diagnostics). For subgrouping in this study, we used the manufacturer-recommended cut-off (negative, < 20.0 IU/mL), consistent with routine clinical practice at our institution. The single assay and cut-off were applied uniformly for internal consistency. Importantly, ACPA status was used for subgroup stratification within RA, not to establish the RA diagnosis itself.

Subjects in the healthy control group were reported as not having any overt disease or adverse symptoms at the time of sample collection. Demographic and clinical data, including the numbers of tender and swollen joints, patient and evaluator global assessments, CRP (mg/L), BMI (kg/m^2^), smoking history, and results for rheumatoid factor (RF, IU/mL) and anti-CCP antibodies were collected from the electronic medical records.

Plasma samples from patients with RA were stored in our ongoing Mayo Clinic Rheumatology Biobank. This biorepository was created for long-term storage of diverse biological samples (e.g., serum, plasma, stool, white blood cells) from patients for use in research. In addition, plasma samples from healthy donors participating in the Mayo Clinic Biobank were used as controls. All methods and procedures were performed in accordance with the Mayo Clinic Institutional Review Board guidelines and regulations. All patients provided written informed consent.

### Proteomic profiling

Plasma proteins were measured with SomaLogic’s SomaScan Assay version 4 (23), which simultaneously targets over 7,000 human proteins including cytokines, growth factors, proteases, and hormones. This platform relies upon protein-capture SOMAmer (Slow Offrate Modified Aptamer) reagents. SOMAmers are based on single-stranded, chemically modified nucleic acids, and are designed to optimize high affinity, slow off-rate, and high specificity to target proteins. In brief, the multiplexed, aptamer-based assay measures the relative binding of target proteins to aptamers in relative fluorescence units (RFUs). After protein concentrations were converted into corresponding DNA aptamer concentrations, abundance levels of proteins were quantified with a DNA microarray.

Data standardization, comprised of normalization, plate scaling, and calibration, was performed on the raw assay data to remove systematic biases after microarray feature aggregation. Global reference standards were established for procedures with controls on each plate (i.e., run). Individual, quality control (QC), and calibrator samples were normalized and calibrated to the established global reference standards (details described in **Supplementary Methods**). In addition, SOMAmer reagents that represent control or non-human analytes were removed, resulting in 7,273 proteins for further analysis. Of note, proteins having the same name but with multiple barcodes (i.e., SeqID) were considered as separate features.

### Metabolomic profiling

Ultra-high-performance liquid chromatography-tandem mass spectrometry (UPLC-MS/MS) using Metabolon Inc.’s Discovery HD4™ platform was performed for untargeted metabolomic profiling. Statistical analyses on untargeted metabolomic data were performed using scaled imputed data provided by Metabolon. Briefly, the raw data were normalized to account for inter-day variation, which is a result of UPLC-MS/MS runs over multiple days (details described in **Supplementary Methods**). The peak intensities were then rescaled to set each metabolite’s median equal to 1. Missing values were then imputed with the minimum observed value of the metabolite across all samples, yielding the scaled imputed data. In addition, metabolites with missing values in over 20% of the entire samples were removed, resulting in 1,061 metabolites remaining for further analysis.

### Identification of phenotype-associated omic features

Omic features (proteins and metabolites) associated with a clinical phenotype (study group) were identified using linear regression analysis coupled with effect size (Cohen’s *d*) determination. These analyses were conducted across two pairs of phenotype comparisons: ACPA– RA vs. controls, and ACPA+ RA vs. controls. To mitigate potential confounding effects, linear regression models were adjusted for sex, age, BMI, smoking history, use of prednisone, use of bDMARDs, and use of csDMARDs.

The linear regression model for each omic feature is described in **Equation 1**, which is:


(1) $$Y=X^{T}\beta +\epsilon$$

where *Y* is the continuous abundance of the omic feature, *X* is the vector of predictor variables (including phenotype indicator and potential confounders), *β* is the vector of coefficients, and *ϵ* is the error term. A feature was considered to be associated with the phenotype (i.e., differentially abundant) if its corresponding coefficient for the phenotype term was statistically significant (*P* < 0.01) and if its effect size was above medium (i.e., Cohen’s |*d*| > 0.5).

For clarification, the use of bDMARDs refers to the prescription use of any of the following: abatacept, adalimumab, certolizumab, etanercept, infliximab, rituximab, or tocilizumab. Similarly, csDMARDs refers to hydroxychloroquine, leflunomide, methotrexate, or sulfasalazine. Individuals with missing smoking history were excluded from models that included smoking as a covariate.

### Functional enrichment of proteins

For a set of proteins, enriched functions defined by Gene Ontology biological process (GOTERM_BP_FAT) annotations were identified using DAVID (version 6.8) (24). Enrichment of a biological process was deemed significant for *P*-values less than 0.05, determined by a modified one-tailed Fisher’s exact test.

### Construction of the phenotype-centric multi-omic network

The phenotype-centric multi-omic network was constructed using a three-pronged approach: network inference, network diffusion, and subnetwork identification. In brief, elastic net regularization was used to infer a network capturing associations (i.e., edges) between 8,343 features (i.e., nodes) across all 120 plasma samples. These features spanned proteomics, metabolomics, demographic characteristics, and clinical phenotypes (i.e., ACPA– RA, ACPA+ RA, and controls), integrating data from all samples across the three study groups. Categorical clinical phenotypes were represented by one-hot encoding for inclusion in the network. Subsequently, a random walk with restart (RWR) diffusion algorithm was applied on the inferred network to prioritize the selection of features most closely associated with the phenotype. The resulting subset of selected features of the subnetwork, delineate those most closely associated with (and thereby most predictive of) the phenotypes. The following sections provide more details on the methodology.

### Inferring a multi-omic network using elastic net

Elastic net regularization is a combination of L1 and L2 regularizations, and is effective when *p* >> *n*, i.e., datasets where the number of features (*p*) significantly exceeds the number of samples (*n*) (25). In our approach, each regression treated an omic feature as the response variable. On the other hand, clinical variables, such as sex, age, BMI, one-hot-encoded smoking status, and one-hot-encoded phenotypes, were not used as responses. Elastic-net regularization identified predictors with non-zero coefficients; these predictors were considered to be associated with the response variable.

An undirected graph was then constructed for each feature, where both response and predictor variables were represented as nodes, and edges indicated connections between the response variable and its predictors with non-zero coefficients. Notably, the clinical phenotype (ACPA– RA, ACPA+ RA, Control) was one-hot encoded into three binary indicators, yielding three phenotype nodes. This process was repeated for all features, resulting in 8,334 individual undirected graphs. Finally, these 8,334 graph models were merged to formulate a single, all-encompassing multi-omic network. The elastic net’s loss function used in this analysis is described in **Equation 2**, which is:


(2) $$\underset{\beta }{argmin}\;\sum_{i=1}^{n}{\left(y_{i}-\beta_{0}-\sum_{j=1}^{p}\beta_{j}x_{ij}\right)}^{2}+\lambda_{1}\sum_{j=1}^{p}|\beta_{j}|+\lambda_{2}\sum_{j=1}^{p}\beta_{j}^{2}$$

where *n* is the number of samples (1 ≤ *i* ≤ *n*; *n* = 120), *p* is the total number of features (1 ≤ *j* ≤ *p*; *p* = 8,341), *y* is the response variable, *x* represents the predictors with the collection of *x* excluding *y*. The hyperparameters *λ_1_* and *λ_2_* satisfy *λ_1_* + *λ_2_* = 1, while the ratio between L1 regularization and L2 regularization falls within 0 < *λ_1_*: *λ_2_* < 1. The elastic net was implemented using R package “glmnet” (v4.1.1). Hyperparameters of the elastic net model were estimated using 10-fold cross-validation, with the optimal values chosen based on the model’s performance in cross-validation. Selection of the best model is guided by the criterion of minimizing the root mean square error.

### Network diffusion using random walk with restart

Random walk with restart (RWR) was used to perform network diffusion on the previously inferred multi-omic network, aiming to identify a phenotype-centric multi-omic network. RWR, widely recognized as a guilt-by-association method, facilitates the exploration of a network’s topology based on the premise that functionally similar nodes are often in close proximity to each other within networks (26). The R package “diffusr”, an implementation of the Markov random walk, was used to simulate network diffusion, as described in **Equation 3**, which is:


(3) $$p^{t+1}=(1-r)A^{\prime }p^{t}+rp^{0}$$

where *p^0^* is the vector of initialized nodes, *t* is a time step, *p^t^* is the vector at the current time step, *p^t+1^* is the vector at the subsequent time step, *A′* is a column-normalized version of the adjacency matrix *A*, and *r* is the restart rate. Elements of *p^0^* are initialized as 1 or 0 to signify the seed node (i.e., sample phenotype) or all other features, respectively; and normalized to ensure the sum of the elements in *p^0^* equals 1. For calculation simplicity, the adjacency matrix *A* only consists of 0 or 1 so that it represents a graph without weighted edges.

RWR was initialized with the seed vector uniformly distributed across the three clinical phenotype nodes, treating phenotypes symmetrically and producing phenotype-proximity scores for all features. The network diffusion process was conducted using the default options of the “diffusr” R package, where the restart rate *r* (i.e., the probability of the random walker returning to the seed node in the next step of the walk) is set to 0.5, and the diffusion is terminated when the L1 norm difference between *p^t^* and *p^t+1^* falls below 1.0 × 10^−4^. Upon completion, the nodes were assigned a “relevance score” reflecting the probability of the random walker being present at the corresponding node. These relevance scores were utilized to rank the network features, with higher scores indicating a stronger association with the phenotype. Through this approach, RWR propagates “importance” throughout the network, highlighting features closest to the seed nodes. Thus, network diffusion effectively prioritizes informative features that might otherwise be masked by less relevant neighbors.

### Selection of features associated with clinical phenotype

Following the RWR-mediated network diffusion, a relevance score is assigned to each feature reflecting its association with the phenotype. These scores are then ranked in descending order to create a hierarchy of feature importance. The top *N* features (e.g., the top 10, top 20, and so on up to all features) are selected from these ranked scores to construct a phenotype-centric network. (This network is termed “phenotype-centric” because its construction starts with the clinical phenotype or study group as the seed nodes, around which the network is built.) The resulting network is a refined subnetwork, originally derived from the broader multi-omic network inferred by elastic net. This subnetwork is composed solely of nodes representing the top *N* features most strongly associated with the phenotype. Of note, the nodes within this phenotype-centric subnetwork are later used as predictors for training a random forest classifier.

### Classification performance of features from the phenotype-centric multi-omic network

A 5-fold cross-validation scheme was performed to evaluate the classification performance of multi-omic features from the aforementioned phenotype-centric network. This evaluation was conducted on the plasma multi-omic profiles from the three study groups (ACPA– RA (*n* = 40), ACPA+ RA (*n* = 40), and controls (*n* = 40)), with the aim of measuring the AUC, accuracy, sensitivity, specificity, positive predictive value, and negative predictive value for clinical phenotype classification. Missing values in smoking history were handled by treating smoking status as a categorical factor with three levels (Never/Former, Current, Unknown), which were one-hot encoded into binary variables for inclusion in the machine learning models.

For each cross-validation fold, the dataset was divided into two segments: a training set comprising 96 plasma samples (32 from each group) and a test set with 24 samples (8 from each group). All steps of the pipeline—including elastic net network inference, RWR-based feature prioritization, and random forest model training—were performed exclusively on the training set. The held-out test set was used only for final evaluation and did not contribute to feature selection, parameter estimation, or model fitting. The top *N* features were selected from this template network (e.g., the top 10, top 20, and so forth up to all features) for training a random forest classifier. The classifier was tasked with predicting the phenotype, e.g., differentiating between ACPA– RA vs. controls and ACPA+ RA vs. controls on a balanced test set comprising 16 samples (8 from each group). Furthermore, the classifier’s ability to distinguish between RA (combining both RA subgroups) and controls was tested on a test set of 24 samples (8 ACPA– RA, 8 ACPA+ RA, and 8 controls).

## Results

### Study design and participant characteristics

This retrospective, observational cohort study consists of a total of 120 participants categorized into three study groups: patients with ACPA– RA (*n* = 40), patients with ACPA+ RA (*n* = 40), and healthy (i.e., non-diseased) controls (*n* = 40). **Table 1**, **Supplementary Table S1**, and **Supplementary Figure S1** provide the demographic and clinical characteristics of the study participants. All three study groups were matched based on subjects’ age, BMI, race, sex, and smoking history. At the time of plasma sample collection (**Materials and Methods**), all RA patients had established disease with a mean age of 57.9 years (min–max range: 32–76 years); and the disease activity of patients varied from remission to high disease activity, with a mean Disease Activity Score 28 using C-reactive protein (DAS28-CRP) (27, 28) of 3.5 (min–max range: 1.5–7.5). Subsets of patients were on treatment with methotrexate (MTX, 51% or 41 of 80), prednisone (25% or 20 of 80), tumor necrosis factor inhibitor biologic disease-modifying anti-rheumatic drugs (TNF*i*-bDMARDs) (14% or 11 of 80), non-TNF*i*-bDMARDs (8% or 6 of 80), or non-MTX conventional synthetic disease-modifying anti-rheumatic drugs (non-MTX csDMARDs) (31% or 25 of 80).

**Table 1:** Demographic and clinical characteristics of study participants.

	ACPA– RA (*n* = 40)	ACPA+ RA (*n* = 40)	Controls (*n* = 40)	*P*-value
Sex (female/male)	28/12	29/11	28/12	1.0
Age (years)				
Mean ± SD [Q1, Q3] Range (min–max)	59.1 ± 10.5 [55.0, 65.0] 32.0–76.0	56.8 ± 10.4 [50.5, 64.3] 35.0–74.0	59.1 ± 10.5 [55.0, 65.0] 32.0–76.0	0.45
Race (*n*, %)				
White	40 (100%)	40 (100%)	40 (100%)	1.0
Disease duration (years)				
Mean ± SD [Q1, Q3] Range (min–max)	2.6 ± 2.8 [0.1, 3.9] 0.1–9.5	2.8 ± 2.6 [0.1, 4.5] 0.0–9.3	N/A	0.66
BMI				
Mean ± SD [Q1, Q3] Range (min–max)	30.7 ± 8.1 [25.4, 34.0] 19.4–58.2	27.7 ± 5.6 [25.2, 30.4] 18.0–43.5	30.2 ± 8.3 [23.7, 34.3] 18.3–51.6	0.27
Smoking history (*n*)				
Current Never/Former Unknown	2 38 0	1 39 0	4 32 4	0.29
ESR (mm/hr)				
Mean ± SD [Q1, Q3] Range (min–max) Unknown	13.1 ± 15.9 [3.8, 16.3] 1.0–73.0 0	13.3 ± 12.8 [4.0, 20.5] 0.0–42.0 2	N/A	0.68
CRP (mg/L)				
Mean ± SD [Q1, Q3] Range (min–max) Unknown	15.3 ± 26.1 [2.9, 10.9] 2.9–113.5 0	6.8 ± 9.4 [2.9, 4.9] 2.9–54.0 1	N/A	0.09
RF (Yes/No)	14/26	28/12	N/A	0.003
DAS28-CRP				
Mean ± SD [Q1, Q3] Range (min–max) Unknown	3.9 ± 1.7 [2.4, 5.1] 1.5–7.5 4	3.1 ± 1.4 [1.7, 4.2] 1.5–6.4 3	N/A	0.07
Treatment (*n*, %)				
Methotrexate Prednisone TNF*i*-bDMARDs^α^ Non-TNF*i*-bDMARDs^β^ Non-MTX-csDMARDs^λ^	19 (48%) 12 (30%) 3 (8%) 4 (10%) 10 (25%)	22 (55%) 8 (20%) 8 (20%) 2 (5%) 15 (38%)	N/A	0.65 0.44 0.19 0.68 0.33

ACPA– RA, anti-citrullinated protein antibody-negative rheumatoid arthritis; ACPA+ RA, anti-citrullinated protein antibody-positive rheumatoid arthritis; Q1/Q3, lower/upper quartile of the interquartile range; ESR, erythrocyte sedimentation rate; CRP, C-reactive protein; RF, rheumatoid factor; DAS28-CRP, Disease Activity Score 28 using C-reactive protein; N/A, not available; ^α^adalimumab, certolizumab, and etanercept; ^β^abatacept, rituximab, and tocilizumab; ^λ^hydroxychloroquine, leflunomide, and sulfasalazine. *P*-values for categorical and continuous variables were obtained using the Fisher’s exact test and Kruskal–Wallis test, respectively.

**Figure 1:**
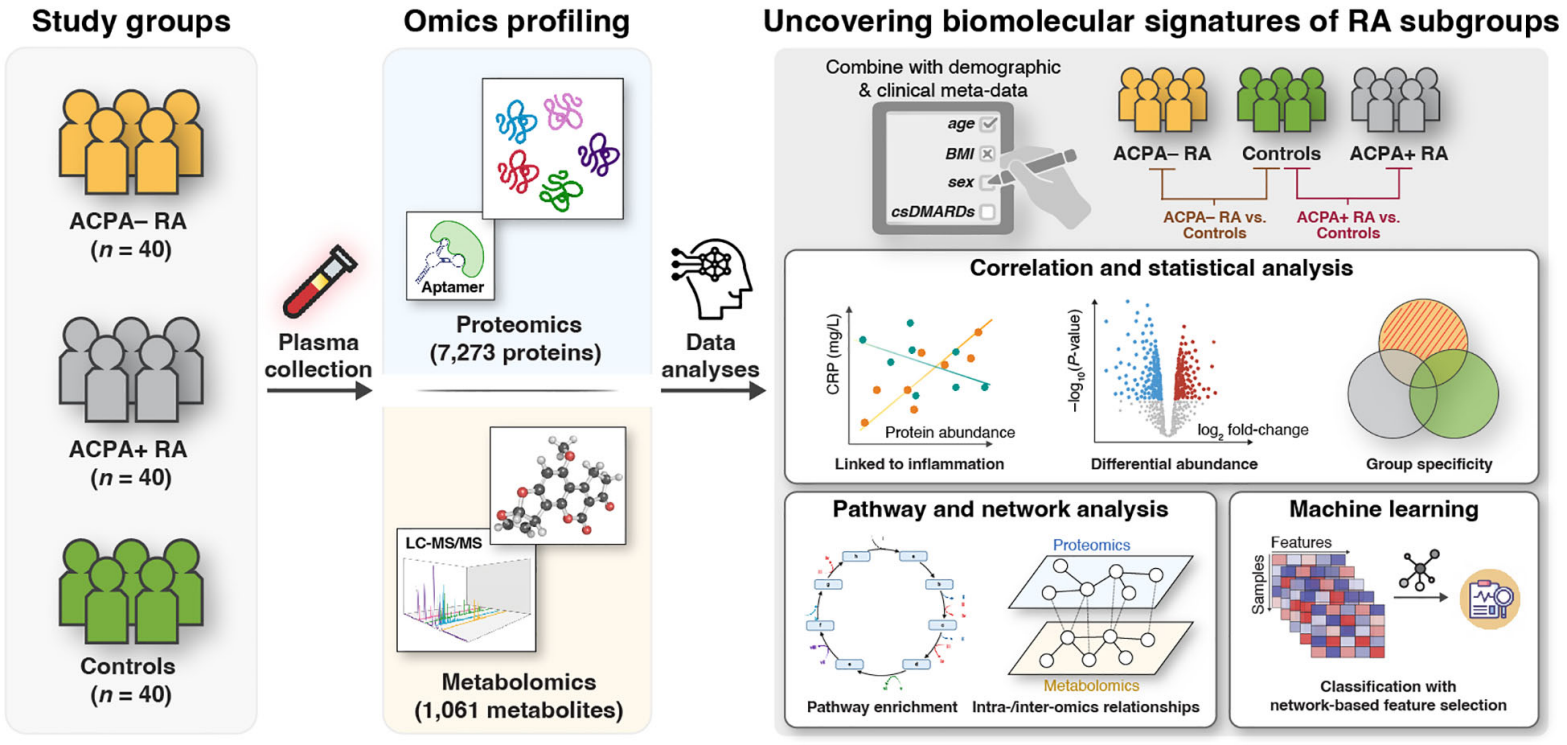
Integrative multi-omic approach to identify RA subgroup-specific biomolecular signatures. This study involves three study groups: Patients with ACPA− RA (*n* = 40), patients with ACPA+ RA (*n* = 40), and healthy (i.e., non-diseased) controls (*n* = 40). Plasma samples were analyzed using untargeted proteomics (7,273 proteins) via an aptamer-based technology (SomaLogic, SomaScan Assay v4); and metabolomics (1,061 metabolites) via LC-MS/MS (Metabolon, Discovery HD4™ platform). Comparative analyses between study groups (ACPA− RA vs. controls and ACPA+ RA vs. controls) were performed to: (1) identify characteristic blood molecules and their associated biomolecular pathways (e.g., immune responses, metabolic reaction pathways); (2) explore intra- and inter-omic relationships; and (3) evaluate the potential of blood molecules to distinguish study groups using machine learning with a network-based feature selection scheme.

An overview of our study design is presented in **Figure 1**. We conducted high-throughput, multi-omic measurements on plasma samples from all 120 study participants. We utilized the SomaScan Assay by SomaLogic (Boulder, CO, USA) for proteomic profiling, and the Discovery HD4™ platform by Metabolon (Durham, NC, USA) for metabolomic profiling (**Materials and Methods**). In total, we analyzed 8,334 biomolecular features, including 7,273 proteins and 1,061 metabolites, to identify associations with the three study groups. Our analyses included statistical comparisons, set-based analyses, and network inference techniques to investigate group-specific patterns. Additionally, we applied a machine learning approach that integrated a network-based feature selection method to develop a computational framework for phenotype prediction. For consistency, we refer to all proteins discussed in the results by their corresponding gene symbols.

### RA subgroup-specific characteristics

**Figure 2:**
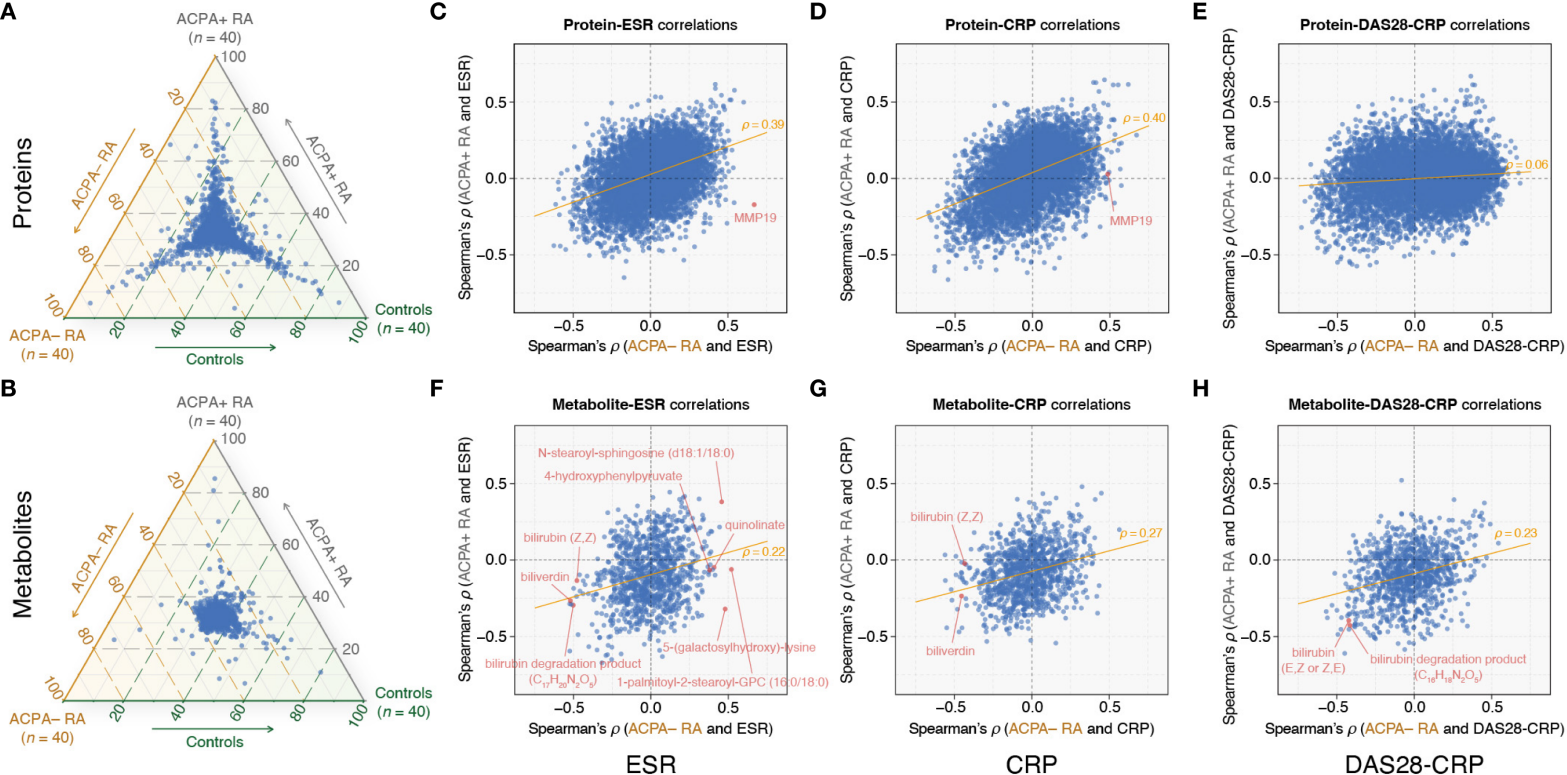
Comparative plasma omics analysis highlights RA subgroup-specific correlations with clinical markers. **(A, B)** Ternary plots showing the distribution of omic feature abundances (mean values) among ACPA– RA, ACPA+ RA, and controls. Each point within the triangle represents a specific protein **(A)** or metabolite **(B)**, with coordinates corresponding to the proportional mean abundances of the feature across the three groups. For example, features clustered near a specific corner are relatively more abundant in that group. **(C–E)** Spearman correlation (*ρ*) analysis between proteins and ESR, CRP, and DAS28-CRP, respectively. **(F–H)** Spearman correlation (*ρ*) analysis between metabolites and ESR, CRP, and DAS28-CRP, respectively. The x-axis shows Spearman’s *ρ* values in the ACPA− RA group, and the y-axis shows Spearman’s *ρ* values in the ACPA+ RA group. The orange trendline indicates the relationship between Spearman’s *ρ* values for ACPA– RA (x-axis) and ACPA+ RA (y-axis). Each point represents the correlation of a specific protein or metabolite with the respective clinical characteristic. ESR, erythrocyte sedimentation rate; CRP, C-reactive protein; DAS28-CRP, disease activity score 28 using CRP.

We next assessed group-wise heterogeneity in biomolecular profiles across ACPA– RA, ACPA+ RA, and controls. In within-omic visualizations (**Figures 2A–B**), proteins showed clearer group differentiation than metabolites, although metabolomic profiles also exhibited detectable separation. Given the substantial disparity in feature dimensionality (proteins: 7,273; metabolites: 1,061), we refrain from cross-omic claims about relative separation magnitude and confine interpretation to within-omic differentiation.

Building on these findings, we investigated the associations (specifically, correlations) between omic features and clinical characteristics within the ACPA– RA and ACPA+ RA subgroups. The scatter plots in **Figures 2C–H** illustrate the correlations of proteins and metabolites with three clinical parameters: the blood inflammatory markers erythrocyte sedimentation rate (ESR) and C-reactive protein (CRP), and DAS28-CRP (a quantitative measure of disease activity) (**Supplementary Tables S2**-**S7**). We observed substantially overlapping correlation patterns between proteins and blood inflammatory markers (ESR and CRP) in both RA subgroups. More specifically, while certain proteins exhibited similar correlation strengths across ACPA− RA and ACPA+ RA, others demonstrated subgroup-specific differences in their relationship with inflammatory markers (**Figures 2C, D**). However, correlations between proteins and DAS28-CRP showed a notable shift, with patterns differing between the two subgroups (**Figure 2E**). In contrast, metabolites in ACPA− RA displayed unique correlation patterns relative to ACPA+ RA, particularly with both blood inflammatory markers and DAS28-CRP (**Figures 2F–H**). These patterns suggest that the relationships between molecular features and clinical markers of inflammation differ between RA subgroups, potentially reflecting subgroup-specific molecular correlates of disease activity. We elaborate below on select examples.

In the ACPA– RA subgroup, our analysis found the Matrix Metallopeptidase 19 (MMP19) protein as having the most positive correlation with ESR (*ρ* = 0.67 and *P* = 2.24 × 10^–6^) (**Figure 2C**; **Supplementary Table S2**). This robust correlation did not extend to the ACPA+ RA subgroup, wherein the correlation between MMP19 and ESR was not significant (*ρ* = –0.17 and *P* = 0.30). Interestingly, MMP19 also correlated positively with CRP (*ρ* = 0.49 and *P* = 1.47 × 10^–3^) in ACPA– RA, but again, this association was absent in ACPA+ RA (*ρ* = 0.03 and *P* = 0.87) (**Figure 2D**; **Supplementary Table S3**). While the specific role of MMP19 in RA is yet to be fully elucidated, it has been previously identified as an autoantigen in the inflamed synovium of RA patients (29). Our findings may position MMP19 as a candidate for further investigation into its mechanistic contributions to the distinct inflammatory profile of ACPA– RA.

Within the ACPA– RA subgroup, our analysis revealed five metabolites (1-palmitoyl-2-stearoyl-GPC (16:0/18:0), 5-(galactosylhydroxy)-lysine, N-stearoyl-sphingosine (d18:1/18:0), 4-hydroxyphenylpyruvate, and quinolinate) that had significant positive correlations with ESR, each with a Spearman’s *ρ* exceeding 0.4 and a corresponding *P*-value below 0.05 (**Figure 2F**; **Supplementary Table S5**). Strikingly, within the ACPA+ RA subgroup, these metabolites either exhibited negative correlations (*ρ* < 0 and *P* < 0.05) or showed no significant correlation (*P* ≥ 0.05). In contrast, within the ACPA– RA subgroup, we identified fourteen metabolites, including biliverdin, bilirubin (Z,Z), and a bilirubin degradation product (C_17_H_20_N_2_O_5_), that demonstrated significant negative correlations (*ρ* < –0.4 and *P* < 0.05) with ESR (**Figure 2F**; **Supplementary Table S5**). In the ACPA+ RA subgroup, however, these correlations were either positive (*ρ* > 0 and *P* < 0.05) or non-significant (*P* ≥ 0.05).

Previous studies in RA have reported negative correlations between bilirubin-derived metabolites and disease activity in RA (30–32). Considering these reports, we investigated the correlation between these metabolites and clinical characteristics in our dataset to identify potential differences between the ACPA– and ACPA+ RA subgroups. Our analysis confirmed that two bilirubin-derived metabolites (bilirubin degradation product [C_16_H_18_N_2_O_5_] and bilirubin [E,Z or Z,E]) exhibited negative correlations with DAS28-CRP (*ρ* < –0.4 and *P* < 0.05) in both the ACPA– and ACPA+ RA subgroups (**Figure 2H**; **Supplementary Table S7**). However, we identified disparate correlations between bilirubin-derived metabolites and the acute phase inflammatory markers (ESR and CRP) in ACPA– and ACPA+ RA. For instance, in the ACPA– RA subgroup, biliverdin and bilirubin (Z,Z) were both negatively correlated with ESR (biliverdin: *ρ* = –0.52 and *P* = 5.11 × 10^–4^; bilirubin (Z,Z): *ρ* = –0.48 and *P* = 1.73 × 10^–3^) and CRP (biliverdin: *ρ* = –0.45 and *P* = 3.28 × 10^–3^; bilirubin (Z,Z): *ρ* = –0.43 and *P* = 5.19 × 10^–3^) (**Figures 2F–G**; **Supplementary Tables S5**, **S6**). Conversely, within the ACPA+ RA subgroup, neither biliverdin nor bilirubin (Z,Z) showed significant correlations with ESR and CRP.

### Plasma proteomic profiling in ACPA– RA and ACPA+ RA

**Figure 3:**
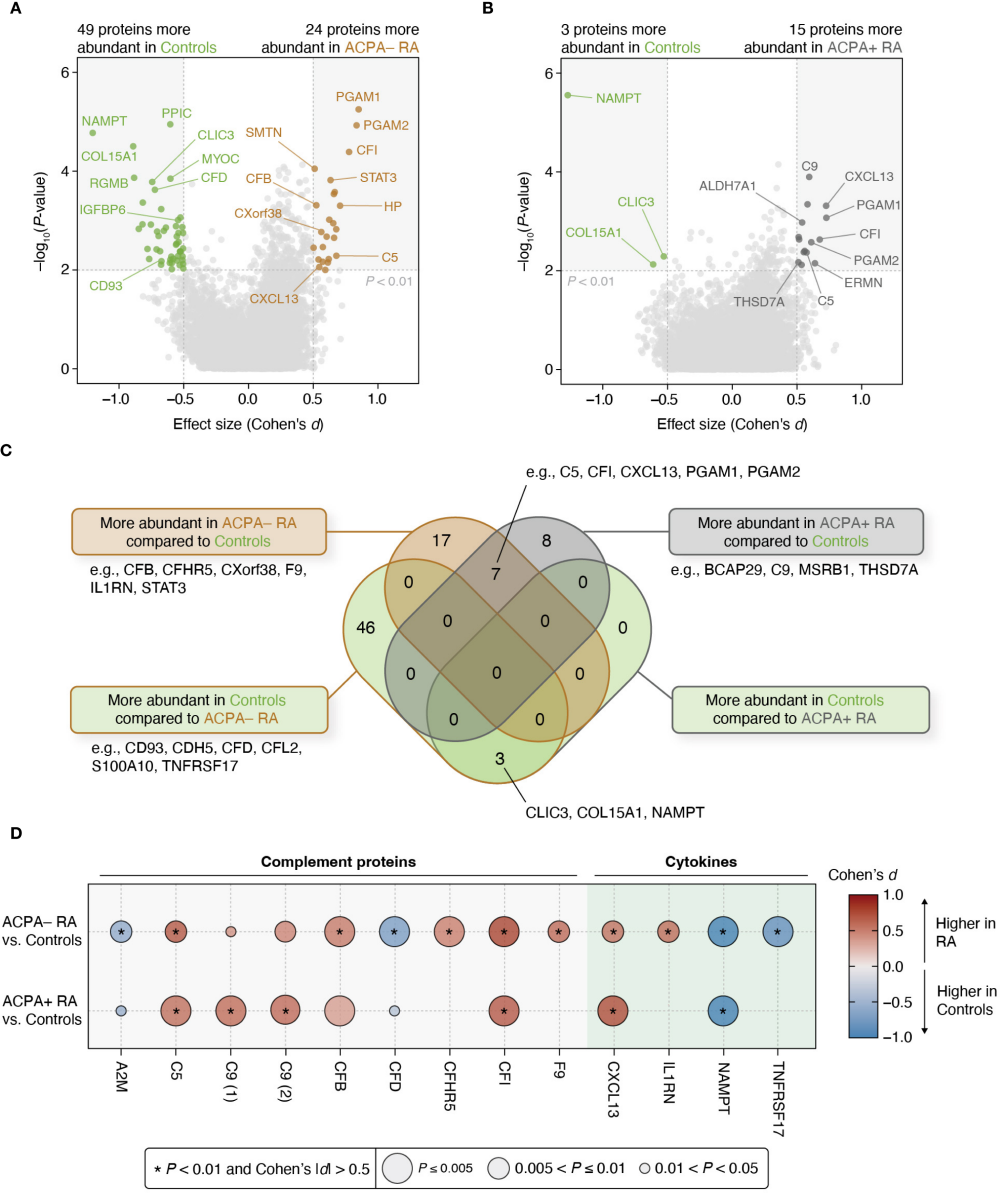
Differential protein abundances reveal contrasting characteristics between ACPA– RA and ACPA+ RA. **(A, B)** Two different pair-wise group comparisons (ACPA– RA vs. controls and ACPA+ RA vs. controls) were conducted to identify differentially abundant proteins. Among the 7,273 measured proteins, those that pass a statistical significance (*P*-value) threshold (i.e., corresponding regression coefficient of *P* < 0.01 in a model adjusted for potential confounders) and an effect size threshold (i.e., Cohen’s |*d*| > 0.5) were considered significantly associated with the corresponding RA subgroup. Age, sex, BMI, smoking history, use of prednisone, use of bDMARDs, and use of csDMARDs were considered as potential confounders. **(C)** Venn diagram illustrating the overlap and uniqueness of differentially abundant proteins found in the pair-wise group comparisons. **(D)** Bubble plot showing complement proteins and cytokines associated with at least one RA subgroup. The size of the bubble represents statistical significance (*P*-value), while the color of the bubble indicates effect size (Cohen’s *d*). Asterisks (*) indicate proteins meeting the study-wide significance threshold (*P* < 0.01 and Cohen’s |*d*| > 0.5). C9 (1) and C9 (2) represent the same complement protein measured by different SOMAmer reagents included in the SomaScan platform.

The identification of distinct correlations in clinical characteristics and biomolecular features between ACPA– and ACPA+ RA motivated us to further investigate the differences in the abundance of individual plasma proteins between study groups. For this, we conducted a differential abundance analysis, selecting proteins with significant group-level differences (*P* < 0.01 for the regression coefficient and Cohen’s |*d*| > 0.5) while controlling for potential confounding factors (i.e., sex, age, BMI, smoking history, use of prednisone, use of bDMARDs, and use of csDMARDs) (**Materials and Methods**). This analysis was structured into two pair-wise group comparisons: ACPA– RA vs. controls, and ACPA+ RA vs. controls. Among 7,273 proteins, we identified 24 proteins with higher abundance, and 49 with lower abundance, in ACPA– RA compared to controls; and ACPA+ RA showed fifteen proteins with higher abundance, and three with lower abundance, than controls (**Figures 3A–B**; **Supplementary Tables S8**, **S9**).

Importantly, seven proteins were commonly elevated in both ACPA– and ACPA+ RA relative to controls, including immune-related proteins such as C5, CFI, CXCL13, PGAM1, and PGAM2. These overlapping proteins may reflect shared systemic inflammatory processes across RA subgroups. Additionally, three proteins (CLIC3, COL15A1, and NAMPT) were found to be consistently lower in both RA subgroups compared to controls, suggesting potentially common pathways of downregulation or depletion associated with RA pathophysiology.

Proteins that were more abundant in each of the two RA subgroups compared to controls (24 proteins in ACPA– RA and fifteen proteins in ACPA+ RA) were both (or commonly) enriched in inflammation-related Gene Ontology (GO) terms, such as “Immune response” (GO:0006955), “Complement activation” (GO:0006956), and “Adaptive immune response” (GO:0002250) (**Supplementary Tables S10**, **S11**). Notably, “Acute-phase response” (GO:0006953) was only enriched in proteins more abundant in ACPA– RA, whereas “Innate immune response” (GO:0045087) and “Pyridine-containing compound catabolic process” (GO:0072526) were only enriched in proteins more abundant in ACPA+ RA.

To further characterize subgroup-specific immune signatures, we examined differences in cytokines and complement proteins using curated lists from ImmPort (33) and the KEGG pathway “Complement and coagulation cascades” (hsa04610). A total of five complement proteins (A2M, CFB, CFD, CFHR5, and F9) and two cytokines (IL1RN and TNFRSF17) were differentially abundant specifically in ACPA– RA, whereas only one complement protein (C9) was differentially abundant specifically in ACPA+ RA (**Figure 3D**). In addition, to assess the robustness of these findings and account for treatment-related confounding, we repeated the analysis in a sub-cohort of treatment-naïve individuals (ACPA– RA, *n* = 14; ACPA+ RA, *n* = 12) using the same thresholds. In this subset, CFB, CFD, CFHR5, and IL1RN remained specific to ACPA– RA, and C9 remained specific to ACPA+ RA, providing further insight into the RA subgroup-specific nature of these immune-related alterations (**Supplementary Tables S12**, **S13**).

### Plasma metabolomic profiling in ACPA– RA and ACPA+ RA

**Figure 4:**
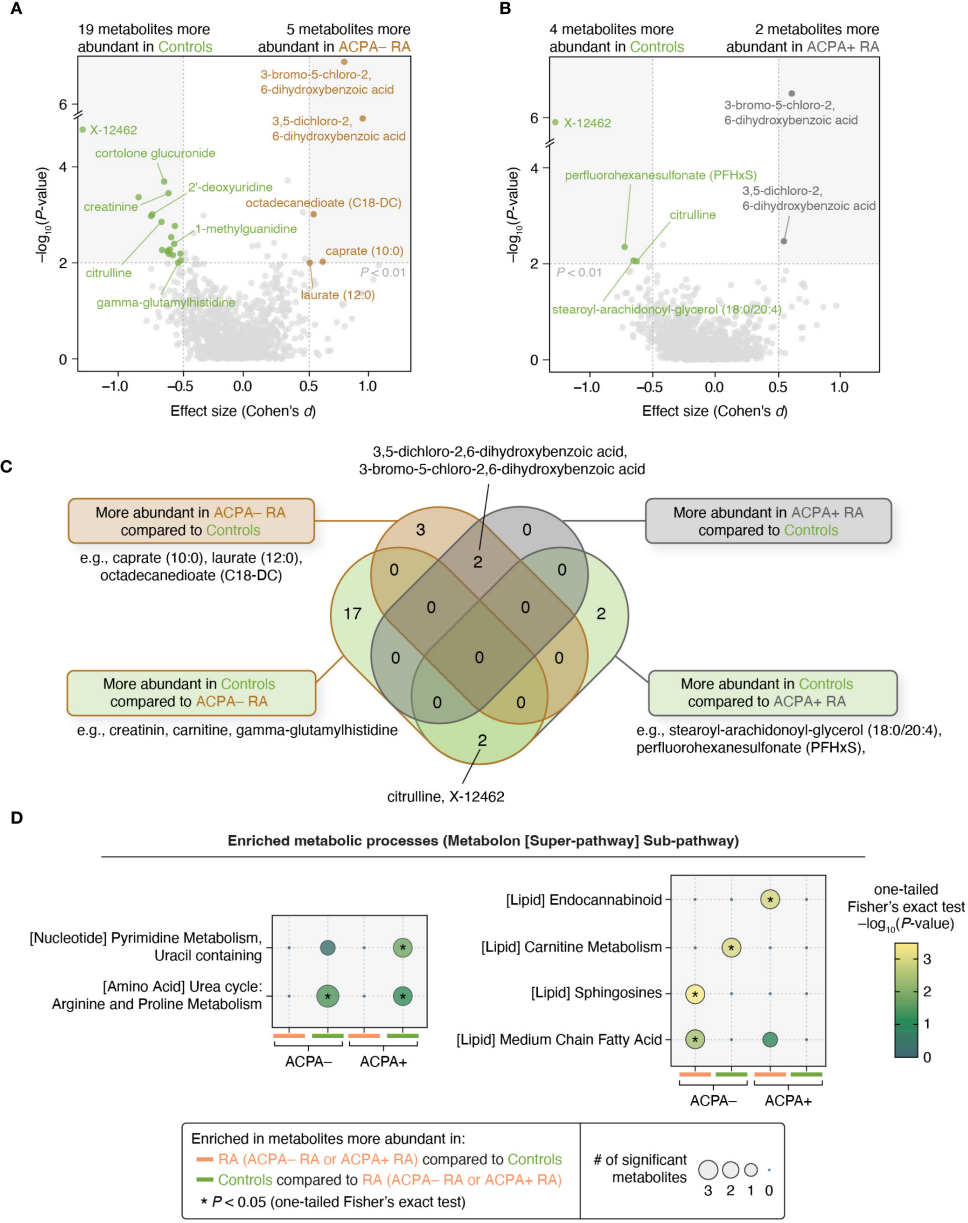
Metabolomic profiles and pathway enrichment analyses reveal differences between ACPA– RA and ACPA+ RA. **(A, B)** Pair-wise group comparisons (ACPA– RA vs. controls and ACPA+ RA vs. controls) were conducted to identify differentially abundant metabolites. Among the 1,061 metabolites analyzed, those demonstrating a significant regression coefficient (*P* < 0.01 in a model adjusted for potential confounders) and an effect size of Cohen’s |*d*| > 0.5 were considered to be differentially abundant. Age, sex, BMI, smoking history, use of prednisone, use of bDMARDs, and use of csDMARDs were considered as potential confounders. **(C)** Venn diagram showing the overlap of metabolites associated with each subgroup. **(D)** Metabolic pathway analysis (using Metabolon’s super-pathway and sub-pathway annotations) revealed distinct pathways enriched in metabolites that were found to be associated with the ACPA– RA and ACPA+ RA subgroups. Statistical enrichment was determined using a modified one-tailed Fisher’s exact test (*P* < 0.05).

We next investigated plasma metabolites to uncover subgroup-specific metabolic traits. For this, we conducted a differential abundance analysis on 1,061 metabolites, using thresholds of *P* < 0.01 for the regression coefficient and Cohen’s |*d*| > 0.5 while adjusting for potential confounders (**Materials and Methods**). We found that in ACPA– RA, five metabolites were significantly more abundant, and 19 were less abundant, than in controls (**Figure 4A**; **Supplementary Tables S14**, **S15**). In ACPA+ RA, there were two metabolites with higher abundance, and four with lower abundance, relative to controls (**Figure 4B**).

Notably, two chlorinated hydroxybenzoic acids (3,5-dichloro-2,6-dihydroxybenzoic acid and 3-bromo-5-chloro-2,6-dihydroxybenzoic acid) were commonly elevated in both RA subgroups compared to controls. In contrast, three metabolites (caprate (10:0), laurate (12:0), and octadecanedioate (C18-DC)) were elevated only in ACPA– RA, suggesting subgroup-specific metabolic differences (**Figure 4C**). To assess the influence of treatment, we repeated the analysis in a sub-cohort of treatment-naïve individuals (ACPA– RA, *n* = 14; ACPA+ RA, *n* = 12), applying the same statistical thresholds. In this subset, caprate (10:0) remained specifically elevated in ACPA– RA, while both chlorinated benzoic acid derivatives continued to be elevated in both RA subgroups compared to controls (**Supplementary Tables S16**, **S17**).

To investigate metabolic pathway-level alterations between study groups, we performed enrichment analysis using a one-tailed Fisher’s exact test on differentially abundant metabolites. Due to the limited number of metabolites meeting our primary significance criteria (*P* < 0.01 and Cohen’s |*d*| > 0.5), we applied a relaxed significance threshold (*P* < 0.05 and Cohen’s |*d*| > 0.5) to ensure sufficient coverage for pathway-level analysis. Under this threshold, we found that in ACPA– RA, twelve metabolites were more abundant and 33 were less abundant than in controls; in ACPA+ RA, seven and seventeen metabolites were more and less abundant than in controls, respectively. For this expanded set of differential features, we observed significant enrichment of pathways related to lipid metabolism (carnitine, sphingosine, and medium-chain fatty acid metabolic pathways) in ACPA– RA; and those related to pyrimidine and endocannabinoid metabolism in ACPA+ RA (**Figure 4D**). Conversely, metabolites that were reduced in abundance in both ACPA– and ACPA+ RA compared to controls were significantly enriched in arginine and proline metabolic pathways, suggesting shared alterations in urea cycle-linked metabolism.

### Multi-omic network inference to elucidate phenotype-associated biomolecular features

**Figure 5:**
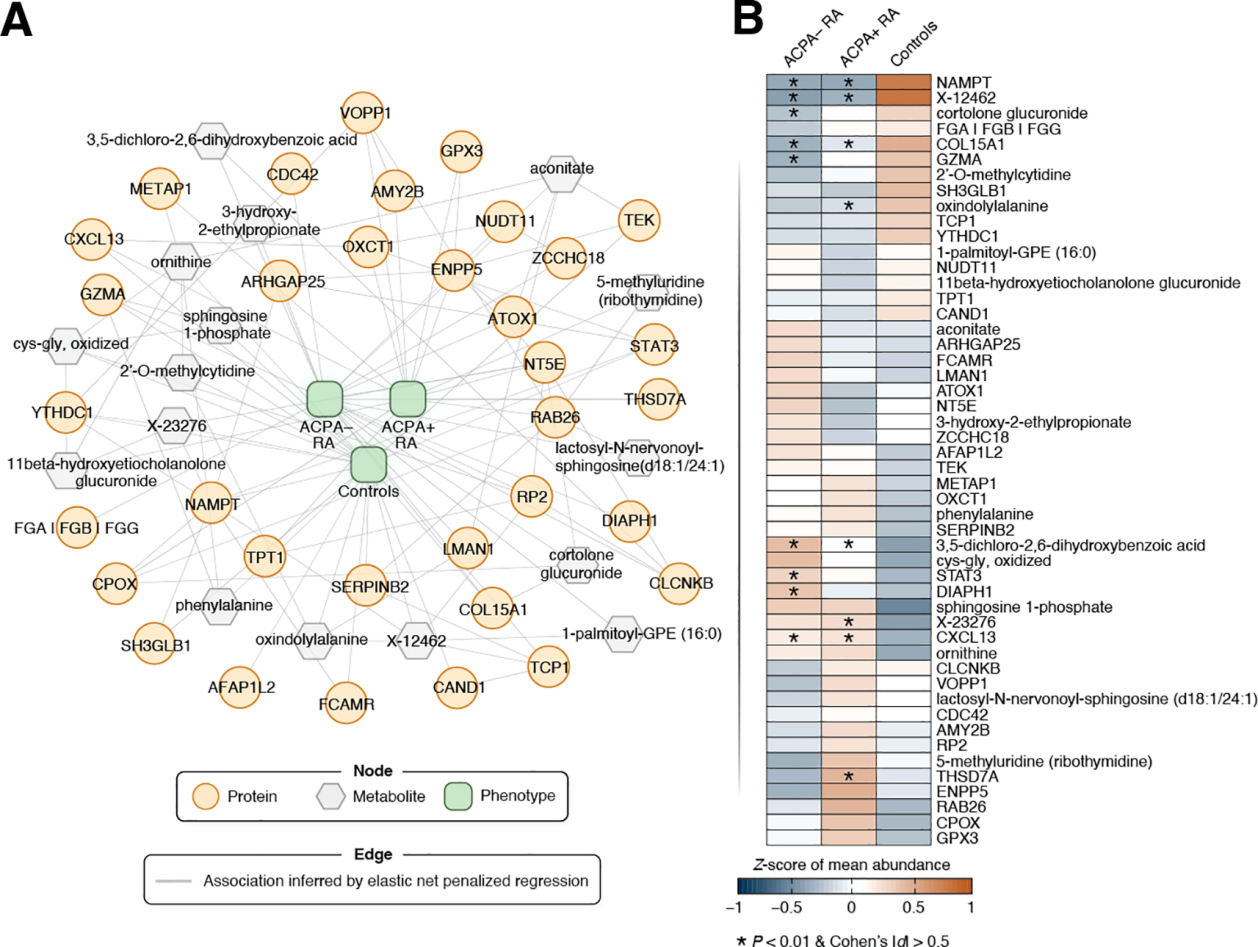
Phenotype-driven multi-omic network of RA-associated biomolecular features. **(A)** This network visualizes predictive associations between omic features and phenotype (i.e., study group), identified using elastic net penalized regression. Biomolecules are shown as nodes, with proteins depicted as orange circles and metabolites as gray hexagons. Associations between biomolecules are represented by edges (gray lines), where links between nodes signify non-zero regression coefficients. All edges in the undirected graph have equal weight. The clinical phenotype (ACPA– RA, ACPA+ RA, Control) was one-hot encoded into three binary indicators, yielding three phenotype nodes in the network (green nodes located in the middle). Omic features closely linked to a clinical phenotype were calculated using a random walk with restart (RWR) approach (see Materials and Methods for a details). Briefly, the phenotype was used as the starting seed for RWR with a 50% chance of being the restart location of the random walker (restart probability *r* = 0.5). For visual clarity, a focused subnetwork comprising the top 50 biomolecules with the closest associations to the phenotype node (based on the network topology) is presented. **(B)** Heatmap showing *Z*-scores of the mean abundance of a network feature in each study group (*n* = 40 samples per group). Features that were previously identified as differentially abundant in RA subgroups compared to controls are marked with an asterisk (*).

We next addressed the integration of proteomic and metabolomic data to uncover cross-omic relationships, which is a task complicated by the complex nature of multi-omic interactions. For this, we applied elastic net penalized regression to infer intra- and inter-omic relationships while incorporating clinical phenotypes (ACPA– RA, ACPA+ RA, and controls) and demographic data (see **Materials and Methods** for details). This analysis produced an extensive multi-omic network comprising 8,341 nodes and 250,092 edges. (Here, nodes represent proteins, metabolites, and demographic characteristics, while edges denote associations between them.) To identify phenotype-relevant features (i.e., those associated with ACPA status in RA), we utilized a network diffusion technique (i.e., random walk with restart) that navigates the network’s topological structure to identify features most closely linked to the phenotype node (**Materials and Methods**). This approach refined the network to a focused subnetwork of 50 biomolecular features most strongly connected to the clinical phenotype (**Figure 5A**). To maintain neutrality and out-of-sample generalizability, we inferred the phenotype-centric network solely from observed data, deliberately excluding external biological priors. This unbiased, data-driven approach is designed to identify parsimonious, low-redundancy cross-omic feature sets that retain high predictive performance and remain amenable to independent validation.

The expression patterns of the prioritized features highlighted subtle, yet discernible, differences among the three phenotypic groups (**Figure 5B**). Interestingly, the classifier features showed only moderate concordance with the univariate signals from our prior statistical analysis; several proteins/metabolites that were not differentially abundant hits received high multivariate importance, consistent with complementary, non-redundant information captured by the integrative model. As is well recognized in multi-omics analyses, classifier and univariate results need not coincide perfectly: a feature can be highly predictive through multivariate dependence (e.g., acting as a network “connector” or reducing redundancy) even if it is not among the strongest by univariate significance, whereas a univariately significant marker may become redundant once correlated markers are modeled jointly.

### Classification of RA phenotypes using network-based biomolecular features

While we applied a covariate-adjusted linear regression strategy for differential testing (interpretability), we also developed a predictive classification pipeline comprising elastic-net network inference (sparse cross-omic associations), phenotype-seeded random walk with restart (feature prioritization), and random forest classification (nonlinear prediction) (**Materials and Methods**). In other words, we extended the network inference approach into a classification framework, leveraging prioritized network features to build predictive models of RA phenotypes. For this, we implemented a classification framework using a 5-fold cross-validation scheme. In each fold’s training dataset, a multi-omic network was first inferred using elastic net penalized regression while integrating phenotypic and demographic data. Subsequently, the random walk with restart algorithm was applied to identify a subset of features most closely associated with the phenotypes. These prioritized features were then used as inputs to a random forest classifier to predict group membership in the test dataset within cross-validation (**Materials and Methods**).

Our network-based machine learning strategy differentiated ACPA– RA patients from controls with an area under the receiver operating characteristic curve (AUC) of 0.92; ACPA+ RA patients from controls with an AUC of 0.93; and RA patients (combining ACPA– and ACPA+ RA) from controls with an AUC of 0.93 (**Table 2**). To provide more granular insight, we report in **Supplementary Table S18** the full set of performance metrics (AUC, accuracy, F1-score, Matthews correlation coefficient), along with misclassification counts (TN, FP, FN, TP), across different subnetwork cutoffs. Notably, our phenotype classification strategy using both multi-omic datasets generally outperformed models trained on single-omic datasets, displaying the value of integrating proteomic and metabolomic data. In addition, across all nine classification tasks (three pair-wise phenotype comparisons × three data modalities), the network-guided approach achieved the highest AUC in most cases and consistently delivered the best classification accuracy (**Supplementary Table S19**). Relative to models trained without feature selection, accuracy improved by up to 28 percentage points; compared with standard univariate filters (e.g., ANOVA F-test, mutual information), the network approach matched or exceeded AUC in most settings and produced markedly higher accuracy. These findings indicate that random-walk-prioritized features capture complementary cross-omic structure and provide a robust foundation for classification. In summary, compared to the diagnostic sensitivity of standardized serological ACPA tests (30–60%), our integrative multi-omic approach could demonstrate a substantial improvement in distinguishing RA patients from controls, particularly in ACPA– RA where clinical diagnosis remains challenging.

**Table 2:** RA classification performance in 5-fold cross-validation.

Classification task^α^	AUC^β^ (mean ± SD)	Accuracy (mean ± SD)	Sensitivity (mean ± SD)	Specificity (mean ± SD)	PPV^γ^ (mean ± SD)	NPV^δ^ (mean ± SD)
ACPA– RA vs. controls						
Multi-omic Proteomic Metabolomic	0.92 ± 0.08 0.91 ± 0.08 0.90 ± 0.12	91.3% ± 7.1% 87.5% ± 8.8% 88.8% ± 12.0%	92.5% ± 6.8% 90.0% ± 10.5% 90.0% ± 10.5%	90.0% ± 16.3% 85.0% ± 10.5% 87.5% ± 15.3%	92.0% ± 12.1% 86.0% ± 9.2% 88.6% ± 13.6%	93.1% ± 6.4% 89.8% ± 9.8% 89.6% ± 11.7%
ACPA+ RA vs. controls						
Multi-omic Proteomic Metabolomic	0.93 ± 0.06 0.85 ± 0.09 0.85 ± 0.12	88.8% ± 5.2% 81.3% ± 12.5% 82.5% ± 8.1%	85.0% ± 10.5% 72.5% ± 22.4% 80.0% ± 11.2%	92.5% ± 11.2% 90.0% ± 16.3% 85.0% ± 20.5%	93.3% ± 9.9% 90.0% ± 14.1% 87.9% ± 16.7%	86.9% ± 8.2% 78.7% ± 14.6% 82.3% ± 10.6%
RA vs. controls						
Multi-omic Proteomic Metabolomic	0.93 ± 0.04 0.83 ± 0.08 0.87 ± 0.13	87.5% ± 5.1% 81.7% ± 6.3% 82.5% ± 6.8%	96.3% ± 5.6% 97.5% ± 3.4% 96.3% ± 5.6%	70.0% ± 16.8% 50.0% ± 17.7% 55.0% ± 30.1%	87.0% ± 6.3% 79.9% ± 6.0% 82.4% ± 10.2%	92.2% ± 10.8% 91.7% ± 11.8% 93.1% ± 10.1%

^α^Three types of classifiers were trained for each classification task: “Multi-omic” used all omic features for network inference, feature selection, and classifier training, while “Proteomic” and “Metabolomic” used solely protein and metabolite components, respectively. The classifier was tested using various cut-offs of subnetwork sizes (i.e., total number of nodes or features) and performance metrics were reported based on the optimal (i.e., highest AUC) subnetwork size. The number of study participants in the ACPA– RA, ACPA+ RA, controls, and total RA (ACPA– RA and ACPA+ RA combined) are 40, 40, 40, and 80, respectively. ^β^AUC, area under the receiver operating characteristic curve. ^γ^PPV, positive predictive value. ^δ^NPV, negative predictive value. Instances where the denominator in the NPV formulas would be zero are excluded from the calculation. **Supplementary Table S18** provides the complete set of performance metrics across different subnetwork cutoffs.

## Discussion

Our integrative multi-omic analysis in plasma revealed that ACPA– RA and ACPA+ RA differ across multiple data modalities, suggesting that these subgroups may have distinct disease biology rather than varying along a single RA spectrum. While prior studies have largely focused on the absence or presence of ACPA as the defining feature of these subgroups, our findings suggest that ACPA– and ACPA+ RA differ at multiple molecular levels. These results not only challenge the conventional view that ACPA– RA is simply a seronegative variant of ACPA+ RA, but also highlight the potential for multi-omic profiling to refine RA classification and improve diagnostic and therapeutic strategies.

ACPA– RA exhibited differentially abundant proteins in immune-related pathways, including acute-phase and complement components—differences that persisted in treatment-naïve individuals. Notably, three complement proteins (CFB, CFD, and CFHR5) were differentially abundant exclusively in ACPA– RA, and these differences persisted in the treatment-naïve sub-cohort. Previous studies have reported that CFB gene expression in synovial tissue correlates with RA disease activity (34), suggesting a possible role for complement activation in local joint inflammation. While our results pertain to protein levels in plasma rather than gene expression in synovial tissue, the elevated CFB levels do align with previously reported transcriptional patterns. While CFB and CFD are both involved in the alternative complement pathway, functional evidence is currently more robust for CFD. In particular, studies using the collagen antibody-induced arthritis (CAIA) murine model have shown that CFD is required for disease induction, as CFD^–/–^ C57BL/6 mice did not develop arthritis (35). Although the animal model does not fully recapitulate human RA, these observations highlight a potential role for alternative pathway components (such as CFD) in shaping inflammatory responses. Our findings raise the possibility that complement activation may be differentially regulated in ACPA– RA, but further mechanistic studies will be needed to establish its relevance to human disease.

In addition to complement components, anti-inflammatory cytokine Interleukin 1 receptor antagonist (IL1RN) was also elevated specifically in ACPA– RA, including in treatment-naïve individuals. While IL1 is a known therapeutic target in RA (36), upregulation of IL1RN in ACPA– RA may reflect a compensatory response to inflammation rather than a deficiency in IL1 inhibition. This raises the possibility that IL1 targeted therapies may have differing relevance across RA subgroups, though further studies are needed to explore this hypothesis.

We acknowledge a few limitations of this study. First, while we utilized cross-validation to assess the performance of our random forest classification models, the ideal scenario would involve testing the classifiers on an independent validation cohort for a more stringent evaluation of our model’s generalizability. Second, the composition of our three study groups (40 persons per cohort) is not a full representation of the broad RA and healthy population, as the majority of our recruited participants were of White race mostly from the Midwest region of the United States. This limits the generalizability of our findings. Future studies with ethnically and geographically diverse cohorts will be necessary for more robust insights regarding ACPA– RA. Third, ACPA– RA itself is a heterogeneous entity that may encompass RF-positive, erosive, or genetically distinct subgroups. Such heterogeneity may obscure biologically meaningful differences between groups, thereby limiting the interpretability of observed molecular patterns. Future multi-omic studies would benefit from stratified designs that account for clinical, serologic, and genetic variation within ACPA– RA to more precisely delineate subgroup-specific signatures. Fourth, heterogeneity in disease activity within the ACPA– and ACPA+ RA subgroups warrants careful interpretation of our results. Our within-subgroup sensitivity analyses indicate that while disease-activity heterogeneity contributes some noise, it is unlikely to be the primary driver of the subgroup-vs.-control differences. Since the independence of ACPA status and disease activity remains uncertain, future studies with more balanced or stratified disease activity distributions will be important to disentangle autoantibody status from disease-activity effects. Fifth, we lose most of the significant “hits” in our statistical analyses after Benjamini–Hochberg multiple hypothesis correction. This could be attributed to multiple factors including a lack of strong differences in biomolecular features between study groups, and the very large number of tests resulting from the high dimensionality of our screening platforms. To mitigate the risk of false discoveries, we carefully designed our cohort selection and applied multiple measures to address possible confounders, such as adjusting for demographic factors and medication effects in our statistical analyses and considering effect size (Cohen’s *d*) as an additional criterion. Sixth, treatment heterogeneity represents a potential confounding factor. Although we adjusted for medication use (bDMARDs, csDMARDs, and prednisone) in our linear regression models, the composition of treatments differed between ACPA– and ACPA+ RA groups. This raises the possibility that some observed biomolecular differences may reflect treatment effects rather than underlying biology. Seventh, although we utilized current state-of-the-art platforms, they capture only a portion of the vast and complex biomolecular landscape in blood. Consequently, this could result in missing important associations with proteins and/or metabolites that could not be profiled. Future advancements in profiling technologies could help mitigate this concern. Eighth, although our machine learning strategy showed high accuracy in distinguishing ACPA– RA patients from controls, it did not account for environmental and lifestyle factors. Not incorporating these factors could potentially hamper how accurately our network inference strategy identifies disease-relevant connections. Therefore, a future study incorporating additional data (e.g., diet, exposome) may enhance the classification performance of our approach. Ninth, while our statistical analyses accounted for BMI, we lacked patient data on metabolic syndrome comorbidities, such as type 2 diabetes and dyslipidemia. The extent to which potential metabolic comorbidities might have impacted our findings remains a topic of future study. Finally, we did not include disease controls such as other inflammatory arthritides. The current analysis focused only on ACPA– RA and ACPA+ RA, raising questions about the specificity of our findings to RA and whether they are applicable to other autoimmune diseases. Future research incorporating disease controls is recommended to validate and extend our findings.

Despite these limitations, our study showcases the transformative potential of multi-omic analyses in understanding subgroup-specific features in RA. By identifying distinct blood protein and metabolite patterns, we provide a strong foundation for future research to validate these findings and explore the biomolecular pathways driving these differences. As a discovery-phase study, our findings highlight the need for translational follow-up, including validation in independent cohorts and assessment of key biomolecules using targeted assay platforms such as ELISA. These efforts could determine whether the identified molecules—particularly those elevated in ACPA– RA—may serve as practical, blood-based biomarkers to supplement existing serologic tests for RA diagnosis. In addition, as we move toward the era of classifying patients based on their biomolecular features (37), we encourage researchers worldwide to build upon our findings using the publicly accessible, de-identified multi-omic datasets we have made available. Through collaborative efforts, we aim to deepen the understanding of seronegative RA, enabling the development of targeted treatment strategies and improving patient outcomes by addressing the unique mechanisms underlying this disease subgroup.

Finally, to bridge our research findings to routine rheumatology practice—and to complement existing ACPA/RF serology with a minimally invasive, plasma-based digital readout—we outline a pragmatic path to implementation. Specifically, to facilitate clinical translation of the proposed classification approach into a clinically applicable tool for individualized patient assessment, we envision (i) locking down a minimal targeted panel with a fixed decision rule or algorithm; (ii) conducting CLIA-concordant analytical validation; and (iii) performing prospective external validation with pre-specified thresholds, calibration, and decision-curve analyses to demonstrate incremental utility beyond ACPA/RF assays. Initial deployment as a laboratory-developed test with EHR-integrated reporting would enable case-level interpretability and ongoing performance monitoring. This staged pathway would prioritize reproducibility, scalability, and real-world impact.

## Data Availability

The datasets presented in this study can be found in online repositories. The names of the repository/repositories and accession number(s) can be found below: https://github.com/hurben/RA_ACPA_multiomics, N/A.
