# Supplementary material for: Integrative multi-omic profiling in blood reveals distinct immune and metabolic signatures between ACPA-negative and ACPA-positive rheumatoid arthritis
Source: Front Immunol. 2025 Oct 29;16:1667662. doi: 10.3389/fimmu.2025.1667662 (PMC12605004; doi:10.3389/fimmu.2025.1667662)
Supplement: Supplementary file 1 [file DataSheet1.zip › Supplementary/Supplementary Information.docx]

Supplementary Information

for

Integrative Multi-omic Profiling in Blood Reveals Distinct Immune and Metabolic Signatures between ACPA-negative and ACPA-positive Rheumatoid Arthritis

Hur *et al*.

**Table of Contents**

**Supplementary Methods**

Data standardization for proteomics (SOMAscan^®^ v4) platform............................................................................3

Data acquisition and normalization for Metabolon UPLC-MS/MS platform..........................................................5

**Supplementary Figure**

Supplementary Figure S1.........................................................................................................................................8

**Data standardization for proteomics (SOMAscan^®^ v4) platform**

Normalization and calibration are routine numerical procedures developed to remove systematic biases in the raw assay data. Normalization is a sample-by-sample adjustment in overall signal within a single plate (run) performed across three non-consecutive steps: Hybridization control normalization, intraplate median signal normalization, and median signal normalization to a reference. Plate scaling and calibration is a SOMAmer^®^ binding reagent-by-SOMAmer^®^ binding reagent adjustment that minimizes between-plate variability. Global reference standards are established for procedures with controls on each plate individual, QC, and calibrator samples are normalized and calibrated to the established global reference standards. Separate calibrator global reference standards are established for each matrix (serum, plasma), and assay shifts or skew from the global reference standards are tracked over time. New global reference standards may be developed in concordance with changes in assay processes, performance, or reagents.

**Hybridization control normalization** was developed to remove systematic biases present in the raw data after slide feature aggregation from a slide-based hybridization microarray for assay readout and quantification. Hybridization control normalization is performed using a set of twelve hybridization control sequences measured independently for each sample array. The procedure is intended to correct for systematic effects on the data introduced during the hybridization readout and results in a single scale factor for each sample that is subsequently applied to the measured signal on all features within a subarray (sample).

**Intraplate median signal normalization** uses all the SOMAmer^®^ reagent signals on a given subarray to remove sample or assay biases that may be due to differences between samples in overall protein concentration, pipetting variation, variation in reagent concentrations, assay timing, and any other source of systematic variability within a single plate. Each SOMAmer^®^ reagent is assigned to one of three dilution sets, scale factors are derived within dilution sets separately, and all SOMAmer^®^ binding reagents within each set are scaled together. Three sample dilutions will result in three independent median signal scale factors for each subarray (sample) in addition to the hybridization scale factor. This step is only applied to calibrator samples.

**Median signal normalization** to a reference occurs on a per-sample basis, wherein a scale factor for a set of SOMAmer^®^ reagents is computed against a reference value generated from a cohort of healthy normal individuals and then aggregated within a dilution. The median of each dilution’s scale factors is then applied to their respective SOMAmer^®^ reagents. This step is applied to QC, buffer, and individual samples.

**Plate scaling and calibration** is accomplished using a number of replicate measurements of a common pooled calibrator sample consistent with the assay sample type for a study. Calibrator samples must be composed of identical sample matrices as the samples that are being calibrated. No protein spikes are added to the calibrator samples—SomaLogic relies solely on the endogenous levels of each analyte within a calibrator sample. Since calibration attempts to correct plate-to-plate variation and such variation can be idiosyncratic for SOMAmer^®^ binding reagents, a unique calibration scale factor is derived for each SOMAmer^®^ binding reagent within the assay. The median of these scale factors is then computed and applied across all SOMAmer^®^ measurements in that plate to account for the total signal difference (plate scale), and the scale factors are subsequently recalculated for each SOMAmer^®^ and applied to all measurements within the set of samples in that plate.

**Acceptance criteria:** Hybridization control and intraplate median signal normalization scale factors are expected to be in the range of 0.4–2.5. The plate scale factor is expected to be between 0.4 and 2.5. The distribution of QC sample ratios is expected to have 85% of individual SOMAmer^®^ reagents in the total array between 0.84 and 1.19 (i.e., less than 15% in the tails of the distribution). Gaussian distributions of scale factors are expected. A report is provided for each study (single plate or set of plates) with the results of the normalization and calibration process.

### **Data acquisition and normalization for Metabolon ultra-high performance liquid chromatography (UPLC)-MS/MS platform**

**Sample accessioning:** Following receipt, samples were inventoried and immediately stored at –80^o^C. Each sample received was accessioned into the Metabolon LIMS system and was assigned by the LIMS a unique identifier that was associated with the original source identifier only. This identifier was used to track all sample handling, tasks, results, etc. The samples (and all derived aliquots) were tracked by the LIMS system. All portions of any sample were automatically assigned their own unique identifiers by the LIMS when a new task was created; the relationship of these samples was also tracked. All samples were maintained at –80^o^C until processed.

**Sample preparation:** Samples were prepared using the automated MicroLab STAR^®^ system from Hamilton Company. Several recovery standards were added prior to the first step in the extraction process for QC purposes. Proteins were precipitated with methanol under vigorous shaking (2 min, Glen Mills GenoGrinder 2000) followed by centrifugation. This step serves to remove proteins, dissociate small molecules bound to proteins or trapped in the matrix, and recover chemically diverse metabolites. The resulting extract was divided into five fractions: two for analysis by two separate reverse phase (RP)/UPLC-MS/MS methods with positive ion mode electrospray ionization (ESI), one for analysis by RP/UPLC-MS/MS with negative ion mode ESI, one for analysis by HILIC/UPLC-MS/MS with negative ion mode ESI, and one sample was reserved for backup. Samples were placed briefly on a TurboVap^®^ (Zymark) to remove the organic solvent. The sample extracts were stored overnight under nitrogen before preparation for analysis.

**Quality control (QC):** Several types of controls were analyzed in concert with the experimental samples: a pooled matrix sample generated by taking a small volume of each experimental sample (or alternatively, use of a pool of well-characterized human plasma) served as a technical replicate throughout the data set; extracted water samples served as process blanks; and a cocktail of QC standards that were carefully chosen not to interfere with the measurement of endogenous biochemical compounds were spiked into every analyzed sample, allowed instrument performance monitoring and aided chromatographic alignment. Instrument variability was determined by calculating the median relative standard deviation (RSD) for the standards that were added to each sample prior to injection into the mass spectrometers. Overall process variability was determined by calculating the median RSD for all endogenous metabolites (i.e., non-instrument standards) present in 100% of the pooled matrix samples.

**Ultra-high performance liquid chromatography-tandem mass spectroscopy (UPLC-MS/MS):** All methods utilized a Waters ACQUITY UPLC and a Thermo Scientific Q-Exactive high resolution/accurate mass spectrometer interfaced with a heated electrospray ionization (HESI-II) source and Orbitrap mass analyzer operated at 35,000 mass resolution. The sample extract was dried then reconstituted in solvents compatible to each of the four methods. Each reconstitution solvent contained a series of standards at fixed concentrations to ensure injection and chromatographic consistency. One aliquot was analyzed using acidic positive ion conditions, chromatographically optimized for more hydrophilic compounds. In this method, the extract was gradient eluted from a C18 column (Waters UPLC BEH C18-2.1×100 mm, 1.7 µm) using water and methanol, containing 0.05% perfluoropentanoic acid (PFPA) and 0.1% formic acid (FA). Another aliquot was also analyzed using acidic positive ion conditions, however it was chromatographically optimized for more hydrophobic compounds. In this method, the extract was gradient eluted from the same aforementioned C18 column using methanol, acetonitrile, water, 0.05% PFPA and 0.01% FA and was operated at an overall higher organic content. Another aliquot was analyzed using basic negative ion optimized conditions using a separate dedicated C18 column. The basic extracts were gradient eluted from the column using methanol and water, however with 6.5 mM ammonium bicarbonate at pH 8. The fourth aliquot was analyzed via negative ionization following elution from a HILIC column (Waters UPLC BEH Amide 2.1×150 mm, 1.7 µm) using a gradient consisting of water and acetonitrile with 10mM Ammonium Formate, pH 10.8. The MS analysis alternated between MS and data-dependent MS^n^ scans using dynamic exclusion. The scan range varied slightly between methods but covered 70–1000 m/z. Raw data files are archived and extracted as described below.

**Bioinformatics:** The informatics system consisted of four major components: the Laboratory Information Management System (LIMS), the data extraction and peak-identification software, data processing tools for QC and compound identification, and a collection of information interpretation and visualization tools for use by data analysts. The hardware and software foundations for these informatics components were the LAN backbone, and a database server running Oracle 10.2.0.1 Enterprise Edition.

**LIMS:** The purpose of the Metabolon LIMS system was to enable fully auditable laboratory automation through a secure, easy to use, and highly specialized system. The scope of the Metabolon LIMS system encompasses sample accessioning, sample preparation and instrumental analysis and reporting and advanced data analysis. All subsequent software systems are grounded in the LIMS data structures. It has been modified to leverage and interface with the in-house information extraction and data visualization systems, as well as third party instrumentation and data analysis software.

**Data extraction and compound identification:** Raw data was extracted, peak-identified and QC processed using Metabolon’s hardware and software. These systems are built on a web-service platform utilizing Microsoft’s .NET technologies, which run on high-performance application servers and fiber-channel storage arrays in clusters to provide active failover and load-balancing. Compounds were identified by comparison to library entries of purified standards or recurrent unknown entities. Metabolon maintains a library based on authenticated standards that contains the retention time/index (RI), mass to charge ratio (*m/z*), and chromatographic data (including MS/MS spectral data) on all molecules present in the library. Furthermore, compound identification is based on three criteria: retention index within a narrow RI window of the proposed identification, accurate mass match to the library ±10 ppm, and the MS/MS forward and reverse scores between the experimental data and authentic standards. The MS/MS scores are based on a comparison of the ions present in the experimental spectrum to the ions present in the library spectrum. While there may be similarities between these molecules based on one of these factors, the use of all three data points can be utilized to distinguish and differentiate compounds. More than 3300 commercially available purified standard compounds have been acquired and registered into LIMS for analysis on all platforms for determination of their analytical characteristics. Additional mass spectral entries have been created for structurally unnamed compounds, which have been identified by virtue of their recurrent nature (both chromatographic and mass spectral). These compounds have the potential to be identified by future acquisition of a matching purified standard or by classical structural analysis.

**Curation:** A variety of curation procedures were carried out to ensure that a high-quality data set was made available for statistical analysis and data interpretation. The QC and curation processes were designed to ensure accurate and consistent identification of true chemical entities, and to remove those representing system artifacts, mis-assignments, and background noise. Metabolon data analysts use proprietary visualization and interpretation software to confirm the consistency of peak identification among the various samples. Library matches for each compound were checked for each sample and corrected if necessary.

**Metabolite quantification and data normalization:** Peaks were quantified using area-under-the-curve. For studies spanning multiple days, a data normalization step was performed to correct variation resulting from instrument inter-day tuning differences. Essentially, each compound was corrected in run-day blocks by registering the medians to equal one (1.00) and normalizing each data point proportionately. Missing values were then imputed with the minimum observed value of the metabolite across all samples (to minimize artificial inflation of low-abundance compounds), yielding the scaled imputed data. In addition, metabolites with missing values in over 20% of the entire samples were removed.

| 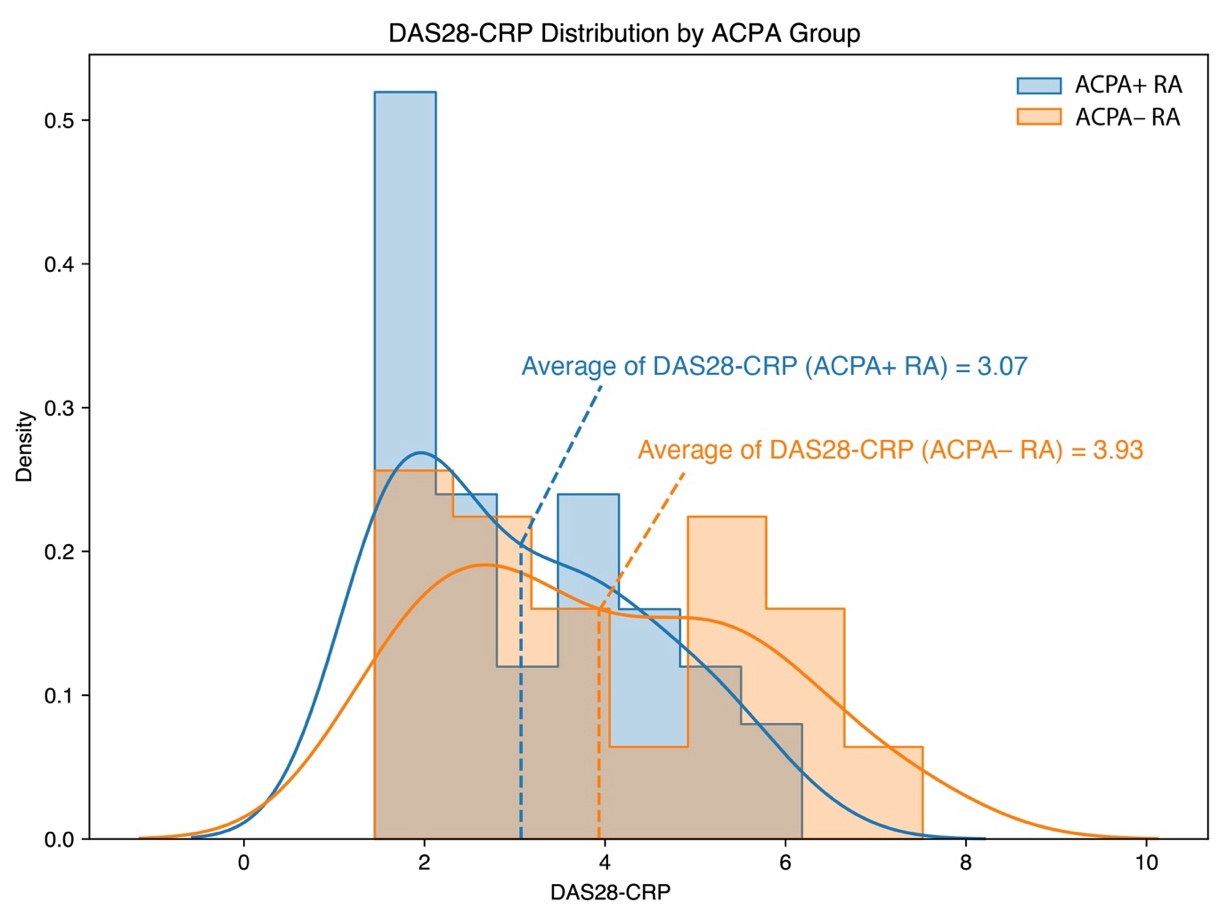 |
| --- |
| **Supplementary Figure S1. Distribution of DAS28-CRP scores in the ACPA– RA and ACPA+ RA subgroups.** Density plots show the distribution of DAS28-CRP scores among patients with ACPA+ RA (blue, *n* = 40) and patients with ACPA– RA (orange, *n* = 40). Vertical dashed lines indicate group averages: 3.07 for ACPA+ RA and 3.93 for ACPA– RA. |
